# *C-reactive protein* gene rs1205 polymorphism is not associated with the risk of colorectal cancer

**DOI:** 10.1042/BSR20170872

**Published:** 2017-08-02

**Authors:** Dilong Fang, Yu Ye

**Affiliations:** Department of General Surgery, Hangzhou Red Cross Hospital/Zhejiang Provincial Integrated Chinese and Western Medicine Hospital, 208 East Road, Hangzhou Ring, Hangzhou, Zhejiang, China

**Keywords:** CRP, CRC, meta-analysis, polymorphism, rs1205

## Abstract

The relationship between *C-reactive protein* (*CRP*) gene rs1205 polymorphism and the risk of colorectal cancer (CRC) has been investigated previously. However, the results were conflicting. In the present study, we assessed whether *CRP* gene rs1205 polymorphism was associated with the risk of CRC by meta-analysis. We searched in PubMed, Embase, and the CNKI databases. Pooled odds ratios (ORs) and 95% confidence intervals (CIs) were calculated. Seven original studies involving 4,181 cases and 10,601 controls analyzed the association between *CRP* gene rs1205 polymorphism and CRC risk. No significant association was found between *CRP* gene rs1205 polymorphism and CRC risk in this meta-analysis. Sensitivity analysis did not draw different findings. Stratification analyses of ethnicity, type of cancer, and genotype method also did not obtain any association between *CRP* gene rs1205 polymorphism and CRC risk. In conclusion, this meta-analysis indicates that *CRP* gene rs1205 polymorphism was not associated with the risk of CRC.

## Introduction

Colorectal cancer (CRC) is the third most common cancer and the fourth most common cancer cause of death globally [1]. The pathogenesis of cancer has not been completely elucidated. However, a significant correlation between inflammation and human cancer was first established almost 27 years ago [2], and inflammatory reactions have received widespread attention in cancer community ever since. Two hypotheses are studied regarding the association between inflammation and cancer. First, the induction hypothesis states that chronic inflammation results in excessive cell proliferation and activation of a cascade of cellular actions that can lead to induction of irreversible DNA damage. Persistent irritation and inflammation subsequently promote these initiated cells, resulting in tumor growth, progression of metastatic disease, and immunosuppression [3]. Second, the immune response of the host is studied as a consequence of tumor growth itself. In both hypotheses, products of inflammatory processes are believed to be biomarkers for cancer [4–6].

C-reactive protein (CRP) is the phenotype acute-phase protein induced by hepatocytes, known as an inflammatory biomarker. The *CRP* gene is located at chromosome 1q21–1q23 consisting of two exons and spans 1.9 kb in length, including 29 single nucleotide polymorphisms (SNPs). CRP is associated with a wide range of diseases, including atherosclerosis and diabetes mellitus [7,8]. Given that cancer is related to several forms of inflammation, CRP levels have also been implicated. Many studies demonstrated that elevated level of CRP was associated with the increased risk of multiple cancers, such as colorectal, esophageal, hepatic, breast, and pancreatic cancer [4,9–14]. Recent data from the European Prospective Investigation into Cancer and Nutrition (EPIC) study showed a positive association between circulating CRP and risk of colon cancer. Therefore, it is reasonable to hypothesize that the *CRP* may be a candidate gene for CRC susceptibility.

Recently, a lot of studies explored the relationship between *CRP* gene rs1205 polymorphism and CRC risk [15–20]. However, the results of these studies were conflicting and inconclusive because of the clinical heterogeneity, different ethnic populations, and small sample sizes. In order to precisely elucidate the genetic role for *CRP* gene rs1205 polymorphism in the development of CRC, we performed a comprehensive meta-analysis to clarify the association between this SNP and CRC risk.

## Materials and methods

### Identification of eligible studies and data extraction

We performed a comprehensive literature search throughout PubMed, Embase, and CNKI databases to retrieve the genetic association studies of CRC. The following terms were used in our searching strategies: ‘c-reactive protein’, ‘CRP’, ‘SNP’, ‘polymorphism’, ‘variant’, ‘cancer’, ‘carcinoma’, and ‘malignancy’. Additional potential omitted studies (such as reference lists of identified studies) have been identified by hand screening. All studies were carefully selected and were up to date as of May 1, 2017. The inclusion criteria for studies were as follows: (1) studies that evaluated the association between *CRP* gene rs1205 polymorphism and CRC risk; (2) studied on human beings; (3) contained genotype data for the calculation of odds ratios (ORs) and 95% confidence intervals (CIs). Related information was carefully extracted from all eligible studies. The following information was extracted from each study: author, year of publication, ethnicity based on the continent of origin of the study population, source of controls (SOC), numbers of cases and controls, and the genotype methods.

### Evaluation of statistical associations

All statistical analyses were performed using the Stata 11.0 software (StataCorp, College Station, TX, U.S.A.). ORs and 95% CIs were used to assess the strength of associations between *CRP* gene rs1205 polymorphism and CRC risk. Stratification analyses were carried out by SOC. *P*<0.05 was considered statistically significant. Multivariate ORs and corresponding 95% CIs between extreme levels of annualized case volume (highest versus lowest) were pooled using a random-effects model, accounting for clinical heterogeneity. Heterogeneity across studies was assessed by using the Q statistic with its *P* value and *I*^2^ statistic [21,22]. Pooled ORs and 95% CIs were calculated in our meta-analysis that was performed using the following genetic models: (1) allele, (2) recessive, (3) homozygous, (4) heterozygous, and (5) dominant. The power of this meta-analysis was calculated with a significant value of 0.05 [23]. Two reviewers independently performed the extraction of data and assessed the quality of study based on the Newcastle–Ottawa Scale (NOS) scores [24]. All disagreements were discussed and resolved with consensus.

### Evaluation of publication bias and heterogeneity

Potential publication bias was assessed by Begg’s and Egger’s linear regression test [25]. *P*<0.05 was considered to indicate statistically significant. We performed sensitivity analysis by omitting each study in turn to determine the effect on the test of heterogeneity and evaluated the stability of the overall results.

## Results

### Characteristics of the included studies

**Figure 1:**
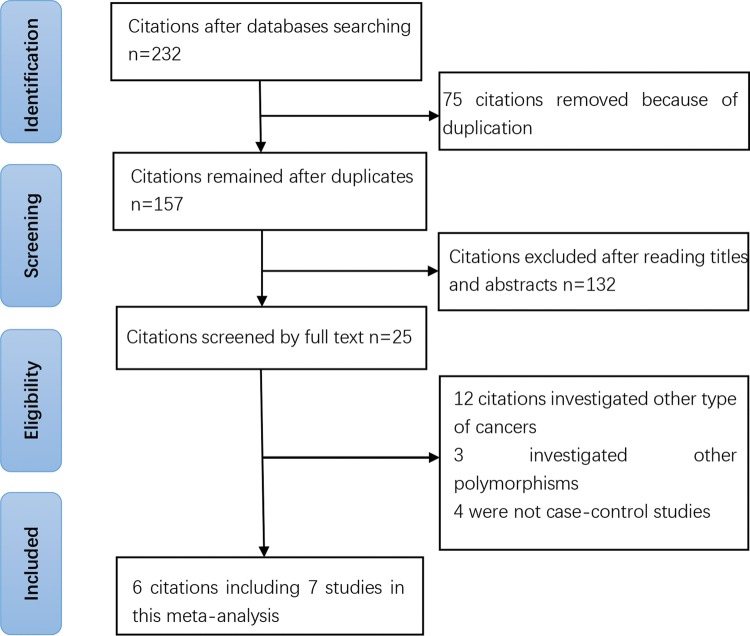
Selection for eligible citations included in this meta-analysis

As showed in Figure 1, we derived 232 citations from the databases of PubMed, Embase, and CNKI. Seventy-five citations were removed due to duplication. Of the 157 remaining citations, 132 were excluded after reading titles and abstracts. Twenty-five citations were excluded after being screened by full text: 12 citations investigated other type of cancers; 3 investigated other polymorphisms; 4 were not case–control studies. The characteristics of included studies are summarized in Tables 1 and 2. The NOS of all included studies ranged from 6 to 8 stars, suggesting that these studies were of high methodological quality.

**Table 1:** Characteristics of included studies

Study	Year	Nationality	Type	Number of cases/controls	Genotype method
Nimptsch et al.	2015	Mixed	CRC	727/727	TaqMan
Yang et al.	2011	China	CRC	421/218	TaqMan
Slattery et al. (CC)	2011	U.S.A.	CRC	1574/1970	Golden Gate assay
Slattery et al. (RC)	2011	U.S.A.	CRC	791/999	Golden Gate assay
Ognjanovic et al.	2010	U.S.A.	CRC	271/539	TaqMan
Tsilidis et al.	2009	U.S.A.	CRC	208/381	TaqMan
Siemes et al. (CRC)	2006	Holland	CRC	189/5767	TaqMan

Abbreviation: CC, colon cancer.

**Table 2:** Characteristics of included studies

Author and year	SOC	Ethnicity	Case			Control			NOS
			CC	CT	TT	CC	CT	TT	
Nimptsch (2015)	PB	Mixed	358	292	71	302	342	81	8
Yang (2011)	PB	Asians	72	197	152	40	111	67	6
Slattery (2011) (CC)	PB	Mixed	700	659	163	882	845	157	6
Slattery (2011) (RC)	PB	Mixed	295	325	79	406	403	92	7
Ognjanovic (2010)	PB	Mixed	55	119	96	146	250	140	7
Tsilidis (2009)	PB	Mixed	99	83	24	167	156	51	6
Siemes (2006) (CRC)	PB	Caucasians	78	92	19	2584	2595	588	7

Abbreviations: CC, colon cancer; PB, population-based; RC, rectal cancer.

### Meta-analysis of *CRP* gene rs1205 polymorphism

**Figure 2:**
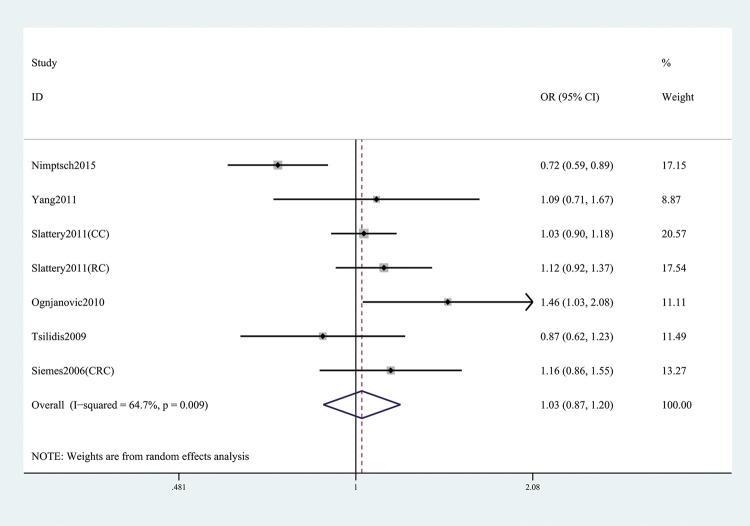
Forest plot shows OR for the associations between rs1205 polymorphism and CRC risk (TT + CT versus CC)

**Figure 3:**
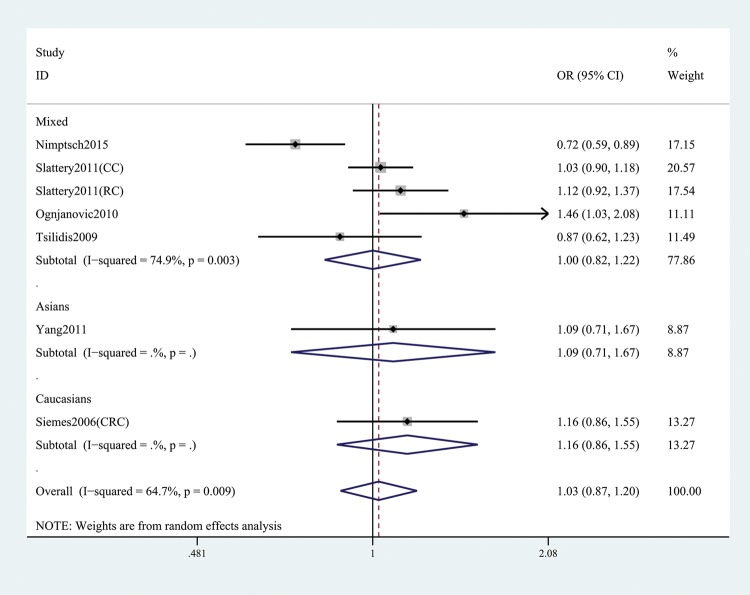
Stratification analysis by ethnicity shows OR for the association between rs1205 polymorphism and CRC risk (TT + CT versus CC)

**Figure 4:**
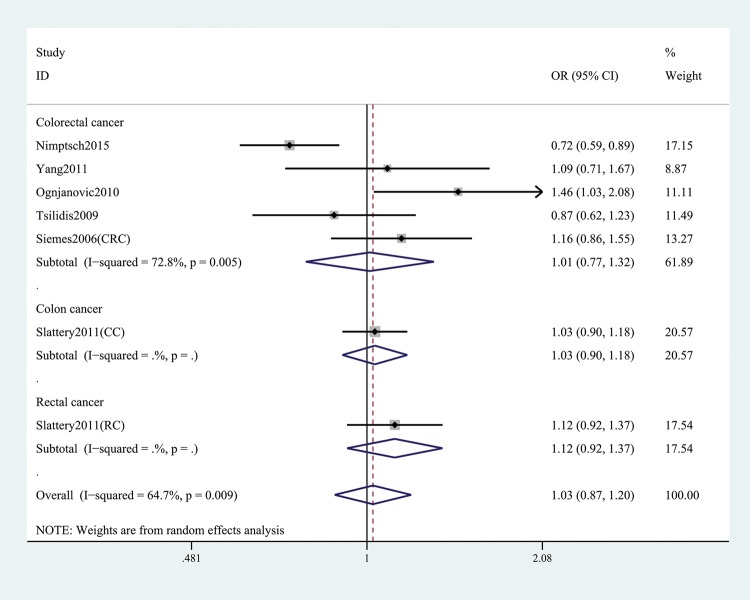
Stratification analysis by type of cancer shows OR for the association between rs1205 polymorphism and CRC risk (TT + CT versus CC)

**Figure 5:**
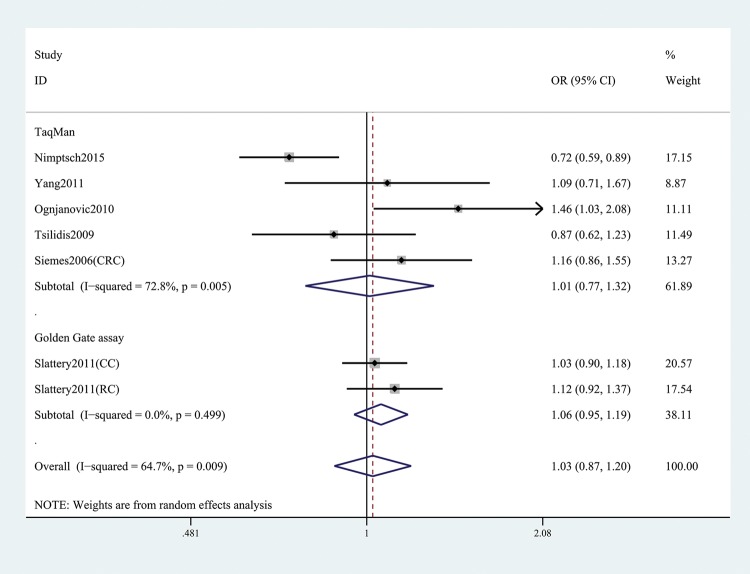
Stratification analysis by genotype method shows OR for the association between rs1205 polymorphism and CRC risk (TT + CT versus CC)

In the general analysis, we found that *CRP* gene rs1205 was not associated with CRC risk (T versus C: OR and 95% CI, 1.05 (0.93, 1.19), *P*=0.421; TT versus CC: OR and 95% CI, 1.14 (0.91, 1.43), *P*=0.257; TT + CT versus CC: OR and 95% CI, 1.03 (0.87, 1.20), *P*=0.758; TT versus CT+CC: OR and 95%CI, 1.16 (0.98, 1.37), *P*=0.078; TC versus CC: OR and 95% CI, 0.99 (0.86, 1.14), *P*=0.866, Table 3 and Figure 2). Stratification analyses of ethnicity (Figure 3), type of CRC (Figure 4), and genotype method (Figure 5) also did not obtain any association between this SNP and CRC risk (Table 3).

**Table 3:** Summary of results of the meta-analysis from different comparative genetic models

Comparison	OR (95% CI)	*P*-value	*P* for heterogeneity	*I*^2^ (%)	Model
T versus C					
Total	1.05 (0.93, 1.19)	0.421	0.002	70.8	Random
Ethnicity					
Mixed	1.03 (0.88, 1.21)	0.695	0.001	79.8	Random
Asians	1.15 (0.91, 1.45)	0.255			
Caucasians	1.08 (0.87, 1.34)	0.489			
Cancer type					
CRC	1.04 (0.85, 1.28)	0.718	0.001	79.3	Random
CC	1.08 (0.97, 1.19)	0.158			
RC	1.09 (0.94, 1.27)	0.240			
Genotype method					
TaqMan	1.04 (0.85, 1.28)	0.718	0.001	79.3	Random
Golden Gate assay	1.08 (0.99, 1.18)	0.064	0.875	<0.001	Random
TT versus CC					
Total	1.14 (0.91, 1.43)	0.257	0.029	57.3	Random
Ethnicity					
Mixed	1.13 (0.84, 1.52)	0.435	0.008	71.1	Random
Asians	1.26 (0.78, 2.04)	0.346			
Caucasians	1.07 (0.64, 1.78)	0.793			
Cancer type					
CRC	1.08 (0.76, 1.54)	0.674	0.014	67.8	Random
CC	1.31 (1.03, 1.66)	0.029			
RC	1.18 (0.84, 1.65)	0.330			
Genotype method					
TaqMan	1.08 (0.76, 1.54)	0.674	0.014	67.8	Random
Golden Gate assay	1.26 (1.04, 1.54)	0.019	0.630	<0.001	Random
TT + CT versus CC					
Total	1.03 (0.87, 1.20)	0.758	0.009	64.7	Random
Ethnicity					
Mixed	1.00 (0.82, 1.22)	0.999	0.003	74.9	Random
Asians	1.09 (0.71, 1.67)	0.694			
Caucasians	1.16 (0.86, 1.55)	0.336			
Cancer type					
CRC	1.01 (0.77, 1.32)	0.929	0.005	72.8	Random
CC	1.03 (0.90, 1.18)	0.632			
RC	1.12 (0.92, 1.37)	0.253			
Genotype method					
TaqMan	1.01 (0.77, 1.32)	0.929	0.005	72.8	Random
Golden Gate assay	1.06 (0.95, 1.19)	0.299	0.499	<0.001	Random
TT versus CT + CC					
Total	1.16 (0.98, 1.37)	0.078	0.140	37.8	Random
Ethnicity					
Mixed	1.15 (0.93, 1.43)	0.205	0.063	55.1	Random
Asians	1.27 (0.90, 1.81)	0.175			
Caucasians	0.98 (0.61, 1.59)	0.949			
Cancer type					
CRC	1.10 (0.86, 1.42)	0.78	0.076	52.7	Random
CC	1.32 (1.05, 1.66)	2.36			
RC	1.12 (0.81, 1.54)	0.70			
Genotype method					
TaqMan	1.10 (0.86, 1.42)	0.78	0.076	52.7	Random
Golden Gate assay	1.25 (1.03, 1.50)	2.32	0.415	<0.001	Random
TC versus CC					
Total	0.99 (0.86, 1.14)	0.866	0.056	51.1	Random
Ethnicity					
Mixed	0.96 (0.81, 1.15)	0.668	0.030	62.7	Random
Asians	0.99 (0.63, 1.55)	0.951			
Caucasians	1.17 (0.86, 1.60)	0.304			
Cancer type					
CRC	0.97 (0.77, 1.22)	0.789	0.040	60.2	Random
CC	0.98 (0.85, 1.13)	0.809			
RC	1.11 (0.90, 1.37)	0.329			
Genotype method					
TaqMan	0.97 (0.77, 1.22)	0.789	0.040	60.2	Random
Golden Gate assay	1.02 (0.91, 1.15)	0.727	0.056	51.1	Random

**Figure 6:**
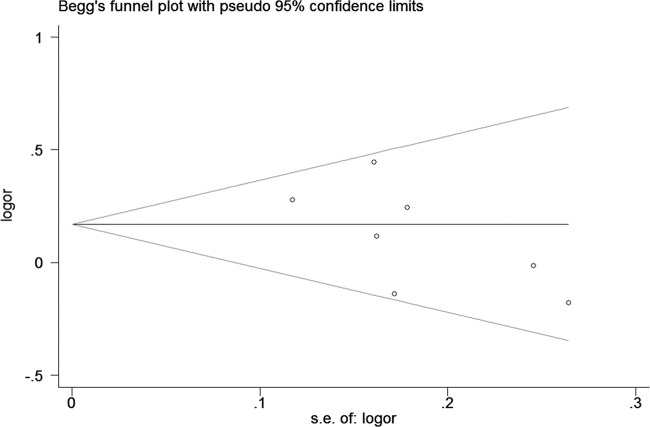
Begg’s tests for publication bias about rs1205 polymorphism and CRC (TT versus CT + CC)

We assessed sensitivity analysis by omitting each study one at a time in every genetic model for rs1205 polymorphism. The pooled ORs for the effects of the SNP on the risk for CRC risk indicated that our data were stable and trustworthy. Both Egger’s and Begg’s tests were used to evaluated the publication bias of this meta-analysis. Our data revealed that there was no obvious publication bias for *CRP* rs1205 polymorphism (Figure 6).

## Discussion

To our best knowledge, this is the first quantitative assessment of the genetic association studies reporting on the relationship between *CRP* gene rs1205 polymorphism and CRC susceptibility. CRP is one of the most common acute-phase proteins induced by hepatocytes. Plasma CRP level may dramatically increase by up to 10,000-fold at the time of acute responses to severe tissue damage or serious infection [26]. Several previous studies reported the association between *CRP* gene rs1205 polymorphism and risk of CRC, but the results were inconsistent [15–20]. This meta-analysis summarized seven case–control studies with 4,181 cases and 10,601 controls, and provided evidence that *CRP* gene rs1205 polymorphism was not associated with CRC risk. Stratification analyses of ethnicity, type of cancer, and genotype method also did not obtain any association between this SNP and CRC risk.

A single study could be underpowered because of sample size, diversity inheritance of the heterogeneous, different ethnicities, clinical heterogeneity, and so on. For instance, Nimptsch et al. [15], Ognjanovic et al. [16], Tsilidis et al. [19], Slattery et al. [18], and Yang et al. [20] reported a significant association between *CRP* rs1205 polymorphism and CRC risk. However, Siemes et al. [17] failed to replicate this association in a study from Netherlands. To overcome these disaccords, we performed this comprehensive meta-analysis to evaluate the association of *CRP* rs1205 polymorphism with CRC risk and different ethnicities. Two meta-analyses [27,28] investigated *CRP* gene rs1205 polymorphism with cancer susceptibility previously. Zhang et al. [27] found no significant association between *CRP* rs1205 polymorphism and the risk of overall cancer. However, in subgroup analysis by cancer type, marginally increased risk was observed in CRC. Geng et al. [28] found that rs1205 polymorphism increased the risk of overall cancer. In addition, stratification analysis of cancer type in their meta-analysis [28] suggested that rs1205 polymorphism was also associated with an increased risk of CRC. We included additional studies and found that this SNP was not associated with CRC risk. Stratification analysis of ethnicity also did not obtain any association between this SNP and CRC risk. It is noteworthy that Zhang et al. [27] did not include two studies [15,20], while Geng et al. [28] did not include four studies [15,17,19,20]. Consequently, the reliability of their conclusions should be interpreted with caution. We believed our meta-analysis has some strengths over previous meta-analyses for the following reasons. First, the present study is the first systematical meta-analysis regarding the association between *CRP* gene rs1205 polymorphism and CRC risk. Second, we identified seven studies [15–20] with larger sample size, including 4,181 cases and 10,601 controls with regard to rs1205 polymorphism. Large sample and unbiased epidemiological studies of predisposition gene polymorphisms could provide insight into the association between candidate genes and diseases. Third, sensitivity analysis indicated that our data about rs1205 polymorphism were trustworthy and robust. Fourth, we conducted stratification analyses of ethnicity, type of cancer, and genotype method (previous meta-analyses did not perform), although no association was obtained. Fifth, the power analysis indicated that our study had a power of 93.2% to detect the effect of rs1205 polymorphism on CRC susceptibility with an OR of 1.14.

Several potential limitations should be addressed in this meta-analysis. First, the heterogeneity of this meta-analysis is high, so the data should be interpreted with caution. Second, due to limited data, we could not conduct further stratification analyses of other potential factors, such as age, gender, smoking, and alcohol consumption. Third, our results were based on unadjusted estimates for confounding factors, which might have affected the final results. Fourth, we could not assess potential gene–gene and gene–environment interactions because of the lack of relevant data. Fifth, the conclusions of some stratification analyses about rs1205 polymorphism should be interpreted with caution due to limited sample size. Sixth, the sample sizes of some stratification analyses were limited. Finally, we cannot examine the association between *CRP* gene rs1205 polymorphism and the clinical manifestations of CRC.

In conclusion, this meta-analysis confirms that *CRP* gene rs1205 polymorphism is not associated with the risk of CRC. Further studies with large sample size is necessary to validate whether *CRP* gene rs1205 polymorphism contribute to CRC susceptibility.

## Abbreviations

CI: confidence interval
CRP: C-reactive protein
NOS: Newcastle–Ottawa Scale
OR: odds ratio
SNP: single nucleotide polymorphism
SOC: source of controls
